# Effects of Dietary Intervention and Education on Selected Biochemical Parameters and Nutritional Habits of Young Soccer Players

**DOI:** 10.3390/nu14183681

**Published:** 2022-09-06

**Authors:** Monika Grabia, Renata Markiewicz-Żukowska, Joanna Bielecka, Anna Puścion-Jakubik, Katarzyna Socha

**Affiliations:** Department of Bromatology, Faculty of Pharmacy with the Division of Laboratory Medicine, Medical University of Białystok, Mickiewicza 2D Street, 15-222 Białystok, Poland

**Keywords:** soccer players, nutritional education, dietary intervention, nutritional habits, nutrients intake, nutritional knowledge, hydration status, young athletes, adolescents, biochemical parameters

## Abstract

In adolescence, the body requires sufficient amounts of adequate nutrients. This is especially important in the case of young athletes, for whom a nutrition plan should be as significant as a proper training plan. The aim of the study was a 17-week follow-up of the effects of individual and group nutrition intervention on changes in eating habits and selected biochemical parameters. 46 young soccer players aged 12–17 from the Soccer Academy in Northeastern Poland completed the study. One group received only individual recommendations, while the other additionally received group nutrition education. As a result of the dietary education, teenagers from the latter group consumed less saccharose (44 g vs. 39.2 g) in favor of digestible carbohydrates (266 g vs. 273 g) and dietary fiber (19.7 g vs. 22.2 g), further emphasizing the health-promoting profile of diets. The amount of fluid consumed (33% vs. 48% above 2 L of water a day) and the habits of the peri-workout hydration routine were also improved. Many of the participants (41%) reported faster regeneration while 26% experienced an overall better well-being. The short-term intervention produced positive results, but nevertheless it is the implementation of long-term dietary improvement schemes involving parents and coaches that should be the direction of future approaches.

## 1. Introduction

The period of intensive growth and development of the body requires provision of adequate amounts of macro- and micronutrients. Nutritional norms are adapted to children and adolescents engaging in moderate physical activity. However, the nutritional needs of teenagers who do sports, often involving one or more daily training sessions, are vastly different. In such cases, not only does proper nutrition influence the maintenance of the optimal rate of physical development of children, but it also improves their sports performance and protects them from the occurrence of injuries related to muscle exhaustion [1,2,3]. Nutrition for a young athlete is not only a challenging task for parents, but also for sports supervisors and athletes themselves. They need to learn the principles of healthy nutrition and then implement them into their daily lives, thereby accelerating recovery after exercise, but also, importantly, reducing the health effects of an improper diet. The frequent lack of adequate education regarding the quality and quantity of meals, contributes to repeated dietary mistakes. Athletes seeking to improve performance use various types of nutritional support products, despite the fact that in a majority of cases it is sufficient to merely modify the standard diet in a judicious manner. Despite the existence of numerous nutritional guidelines, studies show that macronutrient intake by soccer players is probably still insufficient [4,5]. It is necessary to implement nutritional interventions, which are as important as proper training planning [6,7]. Meanwhile, in the literature there is a dearth of studies on nutritional interventions among young soccer players. The adoption of appropriate nutrition education should allow athletes to prepare optimally for competition, and enable them to stay healthy and reach their athletic potential in their further sports careers [1,7,8].

The purpose of this study was to evaluate the effects of individual dietary recommendations and group education on selected nutritional habits and selected biochemical parameters in young soccer players.

## 2. Materials and Methods

### 2.1. Study Group

The research was conducted in the first half of 2021, on a group of 50 young athletes, aged 12–17 years (average age: 15), training in the Soccer Academy in Northeastern Poland. The participants, their parents and sports supervisors had been informed about the purpose of the study and parents had given written consent to their children’s participation in the study. Additionally, the bioethical committee of the Medical University of Bialystok approved the protocol of research (No. R-I-002/587/2019). The study comprised those athletes who, according to their sports supervisors, were making good athletic progress. The presence of a chronic disease and injury was an exclusion criterion for the research.

**Figure 1:**
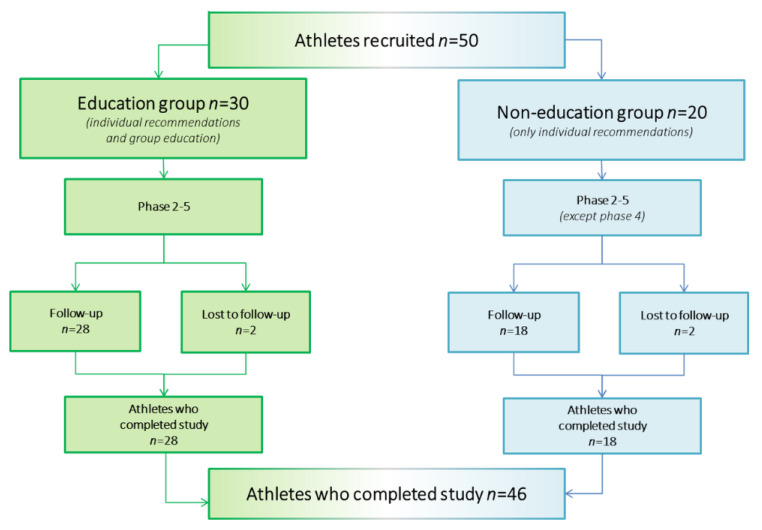
Flowchart of the follow-up process.

The athletes were randomly divided into two groups (Figure 1). The first was an education group (*n* = 30), in which individual recommendations and group education were provided. The members of the second group (*n* = 20) only received individual recommendations. Forty-six participants (*n* = 28 in the education group, *n* = 18 in the other group) were enrolled in a follow-up study after 17 weeks.

### 2.2. Study Design

**Figure 2:**
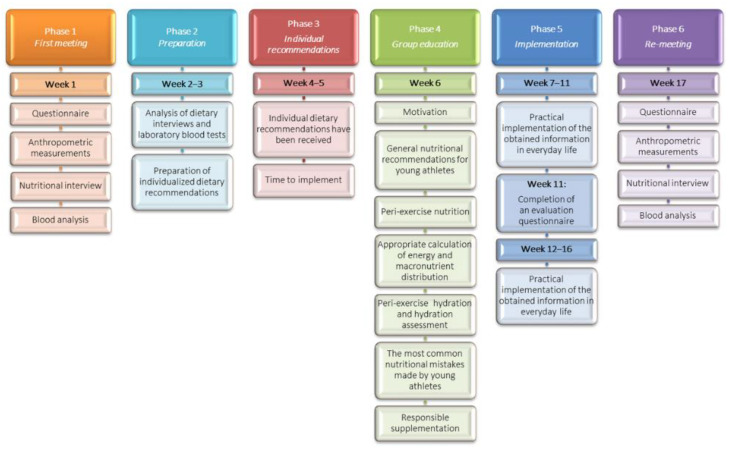
Scheme of the phases of the conducted research.

The study consisted of six phases, as shown in Figure 2.

(1) At the first visit, the participants brought a completed questionnaire and a food diary from the previous three days, which they had been requested to fill out. During the examination, anthropometric measurements and blood sampling for laboratory tests were performed.
(2) The provided food diaries were entered into the “Diet 6” program, which contains Polish databases of nutritional values of food products. After evaluation of dietary nutrient intake and laboratory tests, individual nutrition recommendations were developed for each athlete. These included quantitative information on the intake of macro- and micro-nutrients and their richest sources. Detailed guidance was provided on qualitative dietary analysis and dietary mistakes, with instructions on how to correct them.
(3) Respondents were then given 2 weeks to review the individual nutrition recommendations and attempt to implement them.
(4) Afterwards, a group nutritional education workshop was conducted, which included a 7-part program consisting of sections on motivation, nutritional recommendations for young athletes, peri-exercise nutrition and hydration, supplementation, as well as common nutritional mistakes and improper eating habits. In addition, participants were instructed in how to calculate energy requirements and macronutrient distribution in relation to the training period.
(5) The next phase was to implement the learnt principles and proposed changes over the following 10 weeks. In the middle of this phase (week 11), the participants were given a follow-up questionnaire containing the same questions as previously, but with additional inquiries about the changes implemented and about their subjective perceptions.
(6) At week 17, the examination was repeated as in phase 1.

### 2.3. Nutrition Assessment

Diet assessment was based on a questionnaire that included questions about training sessions, lifestyle, frequency of consumption of specific food groups, and intake of dietary supplements. In addition, questions about subjective assessment of the impact of the intervention on the body were added in the survey conducted at weeks 11 and 17. The Prohealthy-Diet-Index-10 (pHDI-10) and the Non-Healthy-Diet-Index-14 (nHDI-14) were used to qualitatively assess the diets, and were calculated by summing the frequency of consumption of the 10 recommended healthy foods (wholemeal bread; buckwheat groats, oatmeal, whole-grain pasta or other coarse-grain groats; milk; fermented dairy drinks; cottage cheese; dishes made from white meat; fish; dishes made from pulses and lentils; fruits and vegetables) and the nHDI-14 foods (white bread; white rice; plain pasta or small groats; fast foods; meat or flour fried foods; butter; lard; yellow cheeses (including processed cheese, mold cheese); cold cuts, sausages or wieners; dishes made of red meat; sweets; canned meat products; sweetened carbonated or non-carbonated beverages; energy drinks, and alcoholic beverages). Interpretation compares the values: the higher the value of the index, the greater the intensity of favorable or unfavorable health characteristics present (low: 0–33 pts, moderate: 34–66 pts, high: 67–100 pts) [8].

In addition, quantitative intake of macro and micronutrients in the Diet 6 program was estimated using collected dietary interviews from 3 days. Applied daily norms for dietary minerals: sodium (below 1500 mg, 1500–2300 mg, above 2300 mg & 4000 mg), potassium (below 3000 mg, 3000–4500 mg, above 4500 mg), calcium (below 1300 mg, 1300–1700 mg, above 1700 mg) [9], phosphorus (below 1050 mg, 1050–1700 mg, above 1700 mg), and calcium: phosphorus proportion (1:1) [10].

### 2.4. Body Weight and Height Measurements

Body weight was measured fasting, before meals and workout, barefoot, with an accuracy of 0.1 kg on the Inbody 720 device (Inbody, Cerritos, CA, USA). The height of the body in the horizontal position in the Frankfort plane was taken using a height meter, with an accuracy of 0.1 cm (Seca 206, Hamburg, Germany). The measurements were taken in duplicate.

### 2.5. Blood Samples Analysis

Blood samples were collected using vacutainer tubes containing clot activator + gel or anticoagulant K_2_EDTA (Becton Dickinson, France). The material, after centrifugation (10 min and approximately 2000 rpm) and supernatant removal, was transferred into tubes and tested soon after collection. Sodium, potassium and chloride ions were determined by the ion-selective electrode method. Fasting glucose and creatinine were measured using the enzymatic method, and urea using the urease method with an Alinity c analyzer (Abbott Laboratories, Lake Bluff, IL, USA). Osmolality, effective osmolality and osmolal gap were calculated using the formulas below:

$$\mathrm{Osmolality}=1.86\times [{\mathrm{Na}}^{+}\mathrm{mmol}/L]+[\mathrm{glucose}\;\mathrm{mmol}/L]+[\mathrm{urea}\;\mathrm{mmol}/L]\;\mathrm{Effective}\;\mathrm{osmolality}=1.86\times [{\mathrm{Na}}^{+}\mathrm{mmol}/L]+[\mathrm{glucose}\;\mathrm{mmol}/L]\;\mathrm{Osmolal}\;\mathrm{gap}=\mathrm{Osmolality}-\mathrm{Effective}\;\mathrm{osmolality}$$

Applied norms for parameters: sodium (134–143 mmol/L), potassium (3.3–4.6 mmol/L), chloride (97–110 mmol/L), glucose (60–100 mg/dL), creatinine (0.07–1.13 mg/dL), urea (16–41 mg/dL), osmolality (275–305 mOsm/kg H_2_O), and osmolal gap (up to 10) [11].

### 2.6. Statistical Analysis

Statistica software (version 13 PL; TIBCO Software Inc., Palo Alto, CA, USA) was used to perform statistical analysis. The Shapiro-Wilk, the Kolmogorov-Smirnov, and the Lilliefors tests were used to verify the normal distribution of the variables. The Wilcoxon test was applied for the dependent quantitative variables and the Mann-Whitney U test for the independent ones. The significance of the relationship between qualitative dependent variables was assessed with the McNamara test. Values of *p* < 0.05 were considered statistically significant. While designing the study, the minimum required sample size was determined, assuming a maximum error rate (10%) with a fixed confidence level (95%).

## 3. Results

### 3.1. Baseline Characteristics of the Study Cohort

Table 1 provides the characteristics of the study groups. The average age of the respondents was 15 years; most of them lived with their parents (70%) and came from a large city with a population of over 150,000 inhabitants. The majority of the participants were midfielders (41%) and defenders (31%) who trained at least once a day on weekdays (77%) and Saturdays (79%). The average workout on weekdays lasted between 45 and 90 min (57%), or 90–120 min or more (43%), and at weekends usually 45–90 min (56%).

Their dietary model was mainly based on 3–4 meals a day (59%), often eaten regularly (72%). The vast majority (89%) consumed breakfast daily and also had lunch at school (83%). Nearly one in four (24%) participants were on a diet, usually including high-protein (10%) and lactose-free (7%) products.

### 3.2. Results of Nutritional Intervention

Table 2 presents macronutrient intakes estimated on the basis of food diaries collected from both groups at the beginning and end of the study. The main improvements observed in the education group were an increase in digestible carbohydrate (266 g vs. 273 g) and dietary fiber (19.7 g vs. 22.2 g) intake and a decrease in saccharose (44.0 g vs. 39.2 g) consumption.

The qualitative assessment of the collected food diaries revealed that the diets of the athletes in both groups had low intensity of unhealthy and healthy dietary features (Table 3). After the introduction of the recommendations in both groups, there was an increase in the percentage of participants whose diets were characterized by a stronger protective effect of a healthy diet and a moderate effect of an unhealthy diet. In the group that received only individual dietary recommendations, the healthy diet index score increased substantially. In the group receiving additional group education the increase was not as prominent, but the unhealthy diet index decreased (15 pts vs. 13 pts, *p* < 0.01) statistically significantly.

Over 83% of football players used dietary supplements; only 5% of them did it on a doctor’s recommendation. The most frequently chosen were vitamin D (72%), protein supplements (54%), vitamin-mineral complexes, mainly containing vitamin C, B group vitamins, magnesium, potassium, zinc, and iron (35%), fish oil (30%), creatine (11%), carbohydrate supplements (9%), beta-alanine (4%), probiotics, and fiber shakes. Over the duration of the study and with the education provided, the percentage of users of protein supplements decreased (week 11: 30%; week 17: 20%), while that of vitamin-mineral complexes users increased (41% and 48%, respectively).

### 3.3. Hydration Status

One of the most important nutritional problems affecting athletes is inadequate hydration, which can lead to the development of dehydration.

**Figure 3:**
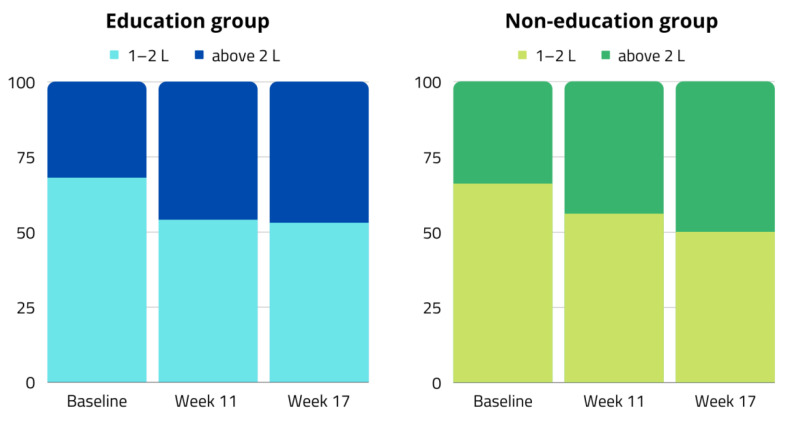
Changes in percentages of daily water consumption by participants noticed during follow-up.

An increase in daily water intake was observed during the control phases in weeks 11 and 17 (Figure 3). Initially, in both groups, consumption of more than two liters of water per day was reported by in 33% of the study subjects. By the last follow-up visit, the percentage had increased by more than 15%.

**Figure 4:**
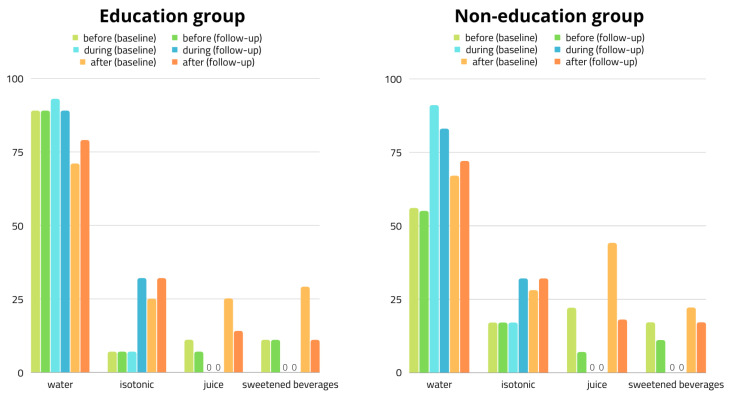
Percentage of participants’ choices of peri-exercise fluid throughout the study.

The most frequently drank peri-workout liquids, apart from water, were isotonics, fruit juices, and sweetened non-carbonated beverages (Figure 4). Less frequently chosen were diluted syrups, flavored waters, sweetened carbonated beverages and energy drinks, and their consumption was further substantially reduced during the study period.

In both groups, high sodium (Na) intake was observed in almost 80% of individuals (Table 4). There was an increase in the percentage of participants who, after the intervention, obtained a potassium intake result within the normal range, compared with the second group, where no difference was noted. In addition, there was a statistically significant increase (15% vs. 41%, *p* < 0.01) in the number of those with adequate calcium (Ca) intake. These changes were apparent in both groups, with an average increase of 12% in each. Moreover, a decrease in the number of persons with insufficient or high phosphorus (P) intake was detected in favor of an increased percentage of results within the normal range. Furthermore, almost all subjects failed to achieve a balanced Ca/P ratio; nevertheless, this percentage was slightly reduced after the implementation of the intervention.

Based on blood laboratory tests, dehydration did not occur; however, there were noticeable percentages of individuals with high concentrations of fasting glucose (41%), potassium (39%), creatinine (27%), and urea (9%). The percentages of teenagers with high fasting glucose and potassium concentrations decreased after the intervention.

### 3.4. Participants’ Subjective Evaluation of Interventions

At the beginning, only 41% of athletes thought they were eating properly (50% in the education group and 28% in the non-education group). Later, as the proposed recommendations were implemented, the percentage of people who said they were eating properly kept rising—at the midpoint, it was 70% (64% and 78%, respectively), and at the last visit 83% (86% and 78%, respectively).

In addition, as time progressed, participants self-assessed their nutritional knowledge (on a scale of 1–5, where 1 was the lowest rating and 5 the highest) consistently higher. At first, 42% of persons chose 3 (46% and 39%, respectively). Over the duration of the study, the vast majority (48% for each) rated themselves as 3 or 4, until finally, at the end of the study, 61% of respondents in both groups assessed their knowledge as 4.

The athletes were also asked to report if the dietary modifications affected their bodies in certain ways. The majority responded that they regenerated a lot faster (26% in the middle of the study and as many as 41% at the end of the study), their overall well-being had improved (24% and 26%, respectively), they found it easier to focus (13% and 4%, respectively), and claimed that they achieved much better results (11% and 9%, respectively).

## 4. Discussion

Soccer is a high-intensity physical activity and thus a high level of physical efficiency is required [12]. Therefore, it is crucial for soccer athletes to apply proper training strategies. However, an equally important, but often overlooked, part of training is a nutritional plan that can be designed and followed if appropriate nutrition education is provided.

One of the most common problems for this group of individuals is an unbalanced ratio of carbohydrates, fats, and proteins as well as vitamins and minerals in their diets [13]. In our study, the average amount of proteins consumed (1.5–1.7 g/kg/day; 18–19% TDEE) and fat (1.0–1.1 g/kg/day; 24–27% TDEE) was appropriate. However, the estimation of protein intake did not take into account additional consumption of protein supplements, in which case the figure would have been much higher. On the other hand, insufficient carbohydrate consumption was observed (4.7–5.2 g/kg/day; 52–56% TDEE). Other authors have shown comparable values in soccer players of similar age. In Briggs et al. [14] the intakes of proteins, fats and carbohydrates were 1.5 g/kg/day, 1.2 g/kg/day, 5.6 g/kg/day, respectively and, in the group studied by Caccialanza et al. [15], 1.5 g/kg/day, 30% TDEE, and 5.0 g/kg/day, respectively.

Indeed, research on soccer players’ nutrition is most often based on a method that estimates the intake of separate nutrients, which is straightforward and provides the crucial information. However, it is worth highlighting that dietary patterns also have an impact on the health and achievements of young athletes [12]. Therefore, a qualitative assessment of diets was conducted using the pHDI-10 and nHDI-14 indexes, revealing the intensity of both correct and inappropriate dietary habits, and found improvements in the nutrition of the studied soccer players. The percentage of athletes whose diets were pro-health increased. In addition, there was a statistically significant reduction (15 pts vs. 13 pts, *p* < 0.01) in the number of points obtained for the index assessing the consumption of unhealthy products in the education group.

At the beginning of the study, 83% of participants reported that they were taking dietary supplements, especially vitamin D (72%) and protein supplements (54%). After individual recommendations and nutrition education had been provided, the overall number of those who were supplementing decreased to 72%, with only 20% taking protein supplements. Other authors [16] reported similar findings; e.g., Manore et al. [17] noted that almost half of the surveyed soccer players took supplements, while one in three used protein supplements.

One of the secondary goals of our study was to obtain information regarding the hydration regimen and hydration status of adolescent soccer players. Analysis showed that, depending on the peri-exercise period, fluid intake might have been insufficient in quantity and that the choice of types of drinks was often suboptimal. This may indicate vulnerability to dehydration although extended blood electrolyte screening tests indicated that hydration levels were not affected. During the trial, deficiencies in the teenagers’ knowledge in this area were eliminated, and their hydration habits improved. Similar results were obtained by Kavouras et al. [18], who educated teenagers at a sports camp about the importance of hydration, providing easy access to water and displaying urine color boards in the toilets. This proved to result in beneficial changes; however, ongoing support and educational instruction from parents and sports supervisors is also desirable. In contrast, a study by Cleary et al. [19] revealed that hydration education alone did not help significantly increase water intake, whereas individualization of needs and obligatory hydration breaks every 20 min had a major impact.

Among our athletes, we observed high Na intake (baseline: 3513 mg/day; follow-up: 3996 mg/day), and a high percentage of individuals (34% 48%, respectively) who consumed more than 4000 mg/day. In addition, insufficient dietary Ca (880 mg/day; 929 mg/day, respectively), with a doubled consumption of P (1581 mg/day; 1600 mg/day, respectively), resulting in an imbalanced Ca:P ratio (0.57; 0.63, respectively) was noted. A comparable result was obtained by Zeng et al. [13], where average Ca intake was around 600 mg/day. Much higher amounts were noted by McCrink et al. [20]—their football players consumed 1081 mg of Ca a day and 2794 mg of Na a day.

Zeng et al. [13] implemented a four-week intervention among 30 elite soccer players, where half received only nutrition education comics, while the other half were given comics and listened to lectures. However, no statistically significant differences in nutrient intake were observed. A study by Fernández-Álvarez et al. [21] suggests that an intervention that uses posters and a web-based application is effective in providing information on healthy eating habits and maintaining diet quality in adolescents attending soccer academies. The need for parental participation in future interventions was also highlighted.

After the 17-week study, the participants’ subjective assessment of their knowledge and implementation of a healthy diet increased substantially in both groups. However, the highest percentage of soccer players who noticed that their nutrition had become healthier was in the education group (86% vs. 78%). It is highly noteworthy that the athletes themselves reported beneficial effects on their bodies (faster regeneration, visibly improved overall well-being, enhanced mental focus, and achievement of better sports results), which could further motivate them to continue improving their nutritional habits.

Among the limitations of our study is that a food diary method was used to assess nutrient intake and thus under- or over-reporting errors might have occurred. Secondly, the study group was relatively small, although we had calculated the minimum required sample size (the maximum error rate was only 10%). Moreover, since this was a follow-up study, the number of volunteers participating in it was definitely limited.

On the other hand, our research has several strengths. First, the drop-out rate was only 8%. Second, the follow-up period was 17 weeks long, thereby making it possible to observe the ongoing progress over time. Third, the dietitian providing individual recommendations was unaware of which participant would take part in the group education, therefore there was no potential risk of influencing the nutritional instructions.

## 5. Conclusions

Our study highlights the importance of educational programs aimed at promoting nutritional awareness among young soccer players. One of the most substantial improvements achieved in the education group was a decrease in the consumption of saccharose in favor of digestible carbohydrates and dietary fiber. The pro-health profile of the diets was reinforced, and the consumption of unhealthy food groups was reduced. Peri-training hydration habits were changed: the amount of fluid consumed increased and quality of peri-training hydration has enhanced. Participants themselves reported that the intervention primarily resulted in faster post-training recovery and improvement of well-being. The short-term intervention yielded successful results. We are convinced that implementation of long-term efforts in the form of nutritional education and involvement of parents and sports supervisors should be one of the priority actions and directions for future endeavors. Additionally, it would be commendable if dietary care was made a regular part of the activity of youth soccer clubs.

## Figures and Tables

**Table 1:** Baseline characteristic of the study cohort.

Parameter	Total (*n* = 46)	Education Group (*n* = 28)	Non-Education Group (*n* = 18)
Age (years)	15 (14–16)	15 (14–16)	15 (14–16)
Body height (cm)	175 (170–181)	177 (168–182)	177 (174–181)
Body weight (kg)	63 (56–71)	62 (56–68)	65 (58–72)
Resides			
With parents At school dormitory	70% 30%	71% 29%	75% 25%
Place of residence			
Village (<10 k inh.) Small city (10–150 k inh.) Large city (≥150 k inh.)	15% 20% 65%	11% 11% 78%	22% 33% 45%
Soccer position			
Goalkeeper Midfielder Striker Defender	11% 41% 17% 31%	4% 50% 14% 32%	22% 28% 22% 28%

Values are expressed as median and interquartile range (Me (Q_1_–Q_3_))or number and percentage of respondents (*n* (%)). Abbreviation: inhabitants (inh.).

**Table 2:** Pre and post intervention changes in macronutrient intake.

Parameter	Education Group (*n* = 28)		Non-Education Group (*n* = 18)	
	Baseline (Week 1)	Follow-Up (Week 17)	Baseline (Week 1)	Follow-Up (Week 17)
	Me (Q_1_–Q_3_)			
Energy (kcal)	2237 (1642–2592)	2107 (1815–2643)	2339 (1881–2845)	2310 (1944–2549)
Protein (%TDEE)	19 (17–21)	18 (18–22)	18 (16–20)	19 (17–22)
Protein (g)	97.6 (84.7–126.5)	105.0 (93.5–125.6)	98.8 (87.8–121.1)	107.5 (81.3–122.3)
Protein (g/kg)	1.7 (1.2–2.0)	1.7 (1.4–2.1)	1.5 (1.3–1.9)	1.6 (1.2–1.8)
Animal protein (g)	71.3 (55.6–84.0)	70.5 (57.6–87.6)	65.3 (57.0–76.6)	68.5 (55.8–78.1)
Vegetable protein (g)	30.5 (22.3–40.3)	29.6 (23.2–43.0)	31.8 (27.9–36.2)	30.1 (24.4–40.8)
Fat (%TDEE)	27.3 (23.4–31.7)	25.5 (22.5–27.5)	24.1 (21.0–29.5)	25.6 (22.7–32.4)
Fat (g)	64.3 (49.0–85.5)	59.4 (47.8–78.4)	65.8 (58.8–74.0)	67.8 (48.6–88.9)
Fat (g/kg)	1.1 (0.8–1.4)	1.0 (0.9–1.1)	1.0 (0.8–1.1)	1.0 (0.8–1.2)
Carbohydrates (%TDEE)	51.8 (48.6–55.2)	54.2 (49.3–58.3)	55.3 (51.9–60.3)	56.3 (50.7–58.1)
Carbohydrates (g)	285 (232–361)	294 (240–417)	333 (243–418)	332 (238–360)
Carbohydrates (g/kg)	5.2 (3.7–6.0)	4.8 (3.8–5.7)	5.1 (4.5–5.8)	4.7 (3.6–5.8)
Digestible carbohydrates (g)	266 (216–338)	273 (223–387)	304 (228–383)	310 (223–334)
Saccharose (g)	44.0 (29.1–70.7)	39.2 (32.6–60.0)	61.2 (43.8–79.5)	67.3 (39.7–83.2)
Dietary fiber (g)	19.7 (15.2–23.8)	22.2 (14.5–27.5)	20.7 (16.4–22.5)	18.1 (14.7–22.9)

Abbreviations: total daily energy expenditure (TDEE). No statistically significant differences between the medians of the dependent variables (week 1 vs. week 17) were detected with the Wilcoxon test.

**Table 3:** Changes in the intensity of healthy and unhealthy diet patterns during the study follow-up period.

Diet Type	Feature Intensity	Education Group (*n* = 28)						Non-Education Group (*n* = 18)					
		Baseline (Week 1)		1st Follow-Up (Week 11)		2nd Follow-Up (Week 17)		Baseline (Week 1)		1st Follow-Up (Week 11)		2nd Follow-Up (Week 17)	
		Me (Q_1_–Q_3_)	%	Me (Q_1_–Q_3_)	%	Me (Q_1_–Q_3_)	%	Me (Q_1_–Q_3_)	%	Me (Q_1_–Q_3_)	%	Me (Q_1_–Q_3_)	%
Healthy	Low	27 (19–38)	61	33 (20–36)	54	30 ^‡^ (20–38)	54	29 (19–37)	72	30 (20–40)	50	39 ^‡^ (24–50)	33
	Moderate		39		46		46		28		50		67
Unhealthy	Low	15 * (13–21)	96	18 * (12–21)	100	13 *^‡^ (11–17)	100	16 (15–26)	89	17 (10–24)	89	19 ^‡^ (16–24)	94
	Moderate		4		0		0		11		11		6

Percentage is expressed as a percentage of respondents who belong to a particular category. * Statistically (*p* < 0.01) significant differences between the medians of the dependent variables (week 1 vs. week 11 and week 11 vs. week 17) were detected using the Wilcoxon test. ^‡^ Statistically (*p* < 0.01) significant differences between the medians of the independent variables (education group vs. non-education group) were detected with the Mann-Whitney U test.

**Table 4:** Diagnosis of dehydration based on dietary intake and blood laboratory test results.

Parameter	Baseline (Week 1)				Follow-Up (Week 17)			
	Me (Q_1_–Q_3_)	Percentage of Respondents (%)			Me (Q_1_–Q_3_)	Percentage of Respondents (%)		
		Below Norm	Norm	Above Norm		Below Norm	Norm	Above Norm
Dietary intake								
Sodium (mg) Potassium (mg)	3513 (2629–4218)	-	22	44 & 34	3996 (2852–4639)	-	2	50 & 48
	3192 (2415–3854)	39	52	9	3389 (2788–3981)	33	54	13
Calcium (mg)	880 (610–1196)	85	15	-	929 (670–1247)	59	41	-
Phosphorus (mg)	1581 (1269–1965)	17	37	46	1600 (1253–1884)	11	50	39
Calcium: Phosphorus	0.57 (0.47–0.64)	98	2	-	0.63 (0.48–0.73)	96	4	-
Blood laboratory test results								
Sodium (mmol/L)	141 (140–142)	-	100	-	141 (140–141)	-	100	-
Potassium (mmol/L)	5 (4–5)	-	61	39	4 (4–5)	-	70	30
Chlorides (mmol/L)	102 (101–103)	-	100	-	103 (102–104)	-	100	-
Glucose (mg/dL)	98 (94–103)	-	59	41	90 (84–95)	-	85	15
Urea (mg/dL)	31 (25–36)	-	91	9	33 (29–37)	-	85	15
Creatinine (mg/dL)	1.0 (0.9–1.1)	-	83	17	1.1 (1.0–1.2)	-	70	30
Osmolality (mOsm/kg H_2_O)	299 (297–301)	-	100	-	296 (294–298)	-	100	-
Effective osmolality (mOsm/kg H_2_O)	294 (292–296)	-	100	-	290 (289–292)	-	100	-
Osmolal gap	5 (4–6)	-	100	-	5 (5–6)	-	100	-

Abbreviation: total daily energy expenditure (TDEE). No statistically significant differences between the medians of the dependent variables (week 1 vs. week 17) were detected by the Wilcoxon test.

## Data Availability

The data presented in this study are available on request from the corresponding author.
